# Cisplatin-Induced Renal Salt Wasting Requiring over 12 Liters of 3% Saline Replacement

**DOI:** 10.1155/2017/8137078

**Published:** 2017-05-10

**Authors:** Phuong-Chi Pham, Pavani Reddy, Shaker Qaqish, Ashvin Kamath, Johana Rodriguez, David Bolos, Martina Zalom, Phuong-Thu Pham

**Affiliations:** ^1^Olive View-UCLA Medical Center, Division of Nephrology and Hypertension, Sylmar, CA 91342, USA; ^2^Olive View-UCLA Medical Center, Division of Hematology and Oncology, Sylmar, CA 91342, USA; ^3^Ronald Reagan UCLA Medical Center, Kidney Transplant, Los Angeles, CA 90095, USA

## Abstract

Cisplatin is known to induce Fanconi syndrome and renal salt wasting (RSW). RSW typically only requires transient normal saline (NS) support. We report a severe RSW case that required 12 liters of 3% saline. A 57-year-old woman with limited stage small cell cancer was admitted for cisplatin (80 mg/m^2^) and etoposide (100 mg/m^2^) therapy. Patient's serum sodium (SNa) decreased from 138 to 133 and 125 mEq/L within 24 and 48 hours of cisplatin therapy, respectively. A diagnosis of syndrome of inappropriate antidiuretic hormone secretion (SIADH) was initially made. Despite free water restriction, patient's SNa continued to decrease in association with acute onset of headaches, nausea, and dizziness. Three percent saline (3%S) infusion with rates up to 1400 mL/day was required to correct and maintain SNa at 135 mEq/L. Studies to evaluate Fanconi syndrome revealed hypophosphatemia and glucosuria in the absence of serum hyperglycemia. The natriuresis slowed down by 2.5 weeks, but 3%S support was continued for a total volume of 12 liters over 3.5 weeks. Attempts of questionable benefits to slow down glomerular filtration included the administration of ibuprofen and benazepril. To our knowledge, this is the most severe case of RSW ever reported with cisplatin.

## 1. Introduction

Cis-diamminedichloroplatinum (CDDP), commonly known as cisplatin, is a chemotherapeutic agent used in the treatment of various cancers including carcinomas, germ cell tumors, lymphomas, and sarcomas. Its antitumor effect is thought to reflect its ability to cross-link with purine bases on DNA, thus interfering with DNA repair mechanisms and leading to DNA damage and tumor cell apoptosis [1]. However, cisplatin is also known to be associated with many adverse effects including gastrointestinal disturbances, hearing loss, allergic reactions, and a multitude of renal problems including electrolyte disturbances, renal tubular acidosis, acute kidney injury with reduced glomerular filtration, Fanconi syndrome, nephrogenic diabetes insipidus, and renal salt wasting (RSW). The pathogenesis of cisplatin-induced renal injuries is thought to be due to renal vasoconstriction and cellular apoptosis and necrosis mediated by altered cell cycle regulation, death receptor signaling, and increased oxidative stress and inflammatory state [1–3].

In a recent literature review, Hamdi et al. analyzed 18 patients who were reported with cisplatin-induced RSW. Patients developed RSW as soon as 12 hours and up to 4 months following cisplatin therapy. Cisplatin dosage used ranged from 80 to 600 mg/m^2^. Renal salt wasting typically only requires transient normal saline (NS) support. With the exception of one patient who required approximately 2.6 L of 3% saline infusion over 2 days, all 17 others only required normal saline support with or without supplemental oral sodium chloride tablets [4, 5]. We herein report a severe RSW case that required 12 liters of 3% saline (6156 mEq Na) over a 3.5-week course, followed by oral sodium chloride supplement for an additional 2 weeks. Preventive and therapeutic considerations for cisplatin-induced RSW will also be discussed.

## 2. Case Presentation

A 57-year-old woman presented with 4 weeks of shortness of breath, dyspnea on exertion, and nonproductive cough. Imaging studies revealed a lung mass extending into the mediastinum with narrowing of the left pulmonary artery and total left upper lobe collapse. Patient was diagnosed with limited stage small cell carcinoma of the lung (SCCL). Initial therapy included cisplatin 80 mg/m^2^ on day 1 and etoposide 100 mg/m^2^ on days 1 to 3 with plan to include radiation at cycle 2. Prior to the administration of cisplatin, patient's serum sodium (SNa) was 134–137 mEq/L. Following the first cisplatin dose, her SNa decreased to 133 mEq/L and 125 mEq/L by 24 and 48 hours, respectively. The acute hyponatremia was initially attributed to the continuous infusion of 5% dextrose half-normal saline in association with presumed lung cancer induced SIADH. However, despite empirical free water restriction, her SNa decreased to 119 mEq/L within 72 hours. Patient developed acute headache, nausea, and fatigue. Renal service was consulted.

Medications included fluticasone nasal spray twice daily, amoxicillin-clavulanic acid 875 mg twice daily, omeprazole 20 mg daily, and empirical intravenous stress dose of hydrocortisone 100 mg q 8 h. As needed medications included metoclopramide 10 mg q 6 h and albuterol 2.5 mg nebulizer treatment q 4 h.

Physical exam revealed blood pressure 123/76 mmHg, heart rate 70 beats per minute, and respiratory rate 18 per minute. Patient was uncomfortable with generalized headaches and nausea but alert and oriented to situation, person, place, and time. Oral mucosa was dry. Heart, lungs, abdomen, and extremities were unremarkable. Neurological exam was nonfocal.

## 3. Results

*Laboratory Findings*. Urine studies revealed osmolality (Uosm) 693 mosm/kg, UNa 205 mEq/L, and potassium (UK) 40 mEq/L in association with increasingly high urine output (average 100–150 mL/h, up to 600 mL/h of urine with UNa + UK up to 265 + 73 mEq/L, resp.). Cisplatin-induced proximal tubular injury with sodium wasting was suspected. Additional laboratory testing revealed glucosuria in the absence of significant serum hyperglycemia (urine glucose 1000 mg/dL, concurrent serum glucose ranged from 117 mg/dL to 141 mg/dL), low-molecular weight proteinuria (0.3 g protein/g creatinine, concurrent albumin/creatinine ratio of 27.7 mcg/mg creatinine), and hypophosphatemia (2.4 mg/dL from baseline of 4.2 mg/dL 1 week previously), all consistent with proximal tubular injury. Serum calcium was also noted to trend downwards from a baseline of 8.78 ± 0.46 mg/dL two days before to 8.25 ± 0.39 mg/dL two days following cisplatin therapy. There was no change noted in serum magnesium. Other routine evaluations for hyponatremia including thyroid stimulating hormone and morning serum cortisol levels were within normal limits.

Patient was started on 3% saline infusion. Her daily effective electrolyte loss (urine sodium and potassium) averaged at 634 ± 183 mEq/d, with a maximum of 1050 mEq.

Patient required approximately 12 L of 3% saline infusion over a 3.5-week period to correct the initial hyponatremia and maintain salt equilibrium prior to gaining sufficient renal recovery for switching to oral sodium supplements. During the worst phase of renal salt wasting, patient required up to 47 g of NaCl or 18.5 g of sodium supplement daily. During the natriuretic phase, attempts to slow her glomerular filtration, thus filtered sodium and potassium load, included ibuprofen 200 mg thrice daily and benazepril 10 mg twice daily (Table 1). It is unclear if our medical therapy ameliorated any renal salt wasting. Addition of fludrocortisone 0.1 mg bid empirically prior to resulting of cortisol did not appear to ameliorate the massive natriuresis. Patient also received intermittent K-phosphate and magnesium supplement during the hospital course. Full clinical renal recovery occurred by 5.5 weeks.

## 4. Discussion

In the setting of malignancy, hyponatremia commonly arises from excess free water intake in association with SIADH, poor solute intake, or, less commonly, adrenal insufficiency due to metastatic disease. Since current patient was initially euvolemic and had no problem with oral intake or known adrenal insufficiency, the diagnosis of SIADH was entertained. Despite empirical free water restriction of one liter a day, patient developed rapid worsening hyponatremia (SNa dropped from 125 mEq/L to 119 mEq/L within eight hours) and acute onset headaches, nausea without vomiting, and clinical evidence of hypovolemia (dizziness and dry oral mucosa) in association with polyuria (up to 4 L a day) of high urine sodium plus potassium content (Table 1). The worsening hyponatremia despite free water restriction and inappropriate and incessant polyuria of high urinary electrolyte content despite volume depletion ruled out SIADH while raising concerns for either cisplatin-induced RSW or undiagnosed adrenal insufficiency. Both SIADH and adrenal insufficiency were eventually definitively ruled out with low vasopressin level (2.4 pg/mL normal range [1.0 to 13.3 pg/mL]) and normal morning cortisol level and lack of response to empirical fludrocortisone therapy, respectively. The diagnosis of cisplatin-induced RSW was thus made.

Cisplatin-induced kidney injury is rare but may lead to acute tubulointerstitial nephritis, tubular necrosis, proximal S3 tubular damage with salt wasting (K^+^, Mg^2+^, Ca^2+^, PO_4_^2−^), Fanconi syndrome, and even nephrogenic diabetes insipidus. Given significant natriuresis and hypophosphatemia, mild tubular proteinuria, and glucosuria in the absence of hyperglycemia, patient was felt to have developed Fanconi syndrome and RSW. Of interest, patient's daily urinary sodium loss was up to 8.9-fold the sodium content of her daily hospital-prepared meals (average sodium content of 2.0 ± 0.2 gm daily), assuming she ate all of her meals. To our knowledge, this is the most severe case of cisplatin-induced RSW ever reported. The reason for severe salt wasting in current case is not known. Patient has no underlying kidney disease and was not receiving any diuretic or nephrotoxic agent that could have potentiated the natriuresis. While we successfully supported patient with high volume 3% saline followed by salt tablets, preventive measures are vital.

Normal saline support during cisplatin therapy is likely the key preventive therapy. The protective effects of NS are thought to involve the ability of the chloride ion concentration to stabilize the CDDP molecule, suppress the formation of CDDP metabolites, and reduce the conversion of CDDP to its aquated products. The latter have higher protein binding capacity and thus greater tissue accumulation and damage [6].

Despite the lack of clinical data, concurrent use of commonly used antioxidants including N-acetylcysteine, vitamin C, and vitamin E has been suggested due to their reported efficacy in animal studies and low toxicity profile. Other clinically available agents considered for renoprotection against cisplatin include salicylates for anti-inflammation, allopurinol for the reduction of reactive oxygen species generation, fibrates for the reduction of free fatty acid accumulation and suppression of apoptosis, and resveratrol for antioxidative, anti-inflammatory, and cytoprotective properties [2].

Alternatively, replacement of cisplatin with carboplatin may be considered in cases with either known contraindication with cisplatin or documented severe cisplatin-induced nephrotoxicity. Nonetheless, it must be recognized that cisplatin has unequivocal superiority over carboplatin in terms of antitumor effect on various malignancies [7]. Subsequent continuation of cisplatin administration can become a difficult clinical decision and must be carefully discussed among the healthcare team and affected patients.

## 5. Conclusions

Clinicians must exert great caution when administering cisplatin due to its severe and potentially life-threatening salt wasting complication. Preventive measures, particularly early normal saline infusion with or without concurrent use of antioxidants, anti-inflammatory agents, or both, must be considered to prevent adverse outcomes. In patients with established severe cisplatin-induced RSW, considerations for an alternative agent may be advisable.

## Figures and Tables

**Table 1:** Hospital course of patient with cisplatin-induced renal salt wasting.

Hospital day	Laboratory findings					Urine volume (mL)	Total (Na + K) urinary loss (mEq/L)	Management	
	SNa (mEq/L)	U(Na + K) (mEq/L)	Uosm (mosm/Kg)	Sosm (mosm/Kg)	SPhos (mg/dL)			Medications	3% NS (mL)
1	138							Cisplatin	
2	133								
3	125, 122, 119, 124, 123	205 + 40	693	262	3.1				300
4	125, 122	212 + 21	907		2.8	3800	885		800
5	124, 127, 126	190 + 38	806		2.7	4050	923		875
6	129, 133, 128, 132	242 + 42	847		2.8	3100	880		750
7	130, 136	270 + 46	832		3	2450	774		500
8	133, 128, 134	250 + 34	903		2.9	2445	694		670
9	132, 122, 124	250 + 32	760		2.8	2460	693		1400
10	132, 131, 130, 135	245 + 15	636		2.7	2485	646		850
11	134, 133	235 + 56	827		2.9	1630	474	Benazepril	650
12	136, 133	212 + 23	810		2.8	2425	569		500
13	133, 129	265 + 42	768		2.7	1950	598		600
14	134, 131	258 + 41	731		3.1	2500	747	Ibuprofen	0
15	132, 130	246 + 73			3.1	1820	580		1000
16	130, 129	246 + 22	637		3.2	1925	515		500
17	129, 138, 126, 130, 131	199 + 87	734		4.1	2375	679		500
18	131, 131	189 + 39			4.5	2850	649		1000
19	138, 138, 135	255 + 54	783		3.1	2200	679		500
20	135	162 + 44	787			3600	741		0
21	135	232 + 54	540			3670	1049		0
22	130, 129, 131					2650	0		0
23	126, 127	225 + 43	660			1750	469		250
24	131, 131, 127	250 + 46	774			1600	473		250
25	130, 127, 131, 134	147 + 69	625			1750	378		250
26	135, 135	197 + 21	528			1800	392		
27	139	145 + 19	404			1700	278		
28	137, 130, 134	218 + 26	675			2225	542		
29	131, 135	209 + 47				2100	537		
30	132					1020			
31	138								
*Total*									*12145*

SNa: serum sodium; U(Na + K): urine sodium plus potassium; Uosm: urine osmolality; Sosm: serum osmolality; SPhos: serum phosphorus; NS: normal saline.
